# Molecular docking of danuglipron uncovers potential crossovers between GLP-1R and the endocannabinoid system

**DOI:** 10.17912/micropub.biology.001690

**Published:** 2025-07-24

**Authors:** Kiersten A Dailey, Lillian N Schneider, Ruben C Petreaca, Ryan J Yoder

**Affiliations:** 1 Biology program, The Ohio State University at Marion, Marion, Ohio, United States; 2 Molecular Genetics, Cancer Biolgoy, James Comprehensive Cancer Center, The Ohio State University at Marion, Marion, Ohio, United States; 3 Chemistry and Biochemistry, The Ohio State University at Marion, Marion, Ohio, United States

## Abstract

The targeting of the G-protein coupled receptor (GPCRs) glucagon-like-peptide-1-receptor (GLP-1R) by weight loss medications has become extremely prevalent due to the effectiveness of a class of GLP-1R agonists. Also, interest in cannabinoids, such as delta-9-tetrahydrocannabinol (THC), has risen independently of GLP’s newfound fame due to the relaxation of legal hurdles across the nation to recreational cannabis usage. THC interacts with receptors in the endocannabinoid system, with the major ones being cannabinoid receptor 1 (CB1) and cannabinoid receptor 2 (CB2), both GPCRs. As these GPCR targets are becoming increasingly of interest due to these independent pathways, this study aimed to identify potential crossover between the endocannabinoid system and the GLP-1R system through molecular docking experiments. This was done using endogenous (2-AG and anandamide) and exogenous (THC) ligands of the endocannabinoid system , along with a proposed small oral agonist (danuglipron) of GLP-1R. Results indicated that danuglipron, a small GLP-1R agonist, had a higher binding affinity for CB1 and CB2 than any of the endogenous or exogenous ligands of the endocannabinoid system, suggesting the potential for cross-reactivity.

**Figure 1. The docking results of CB1 and GLP-1R with ligand selection:**
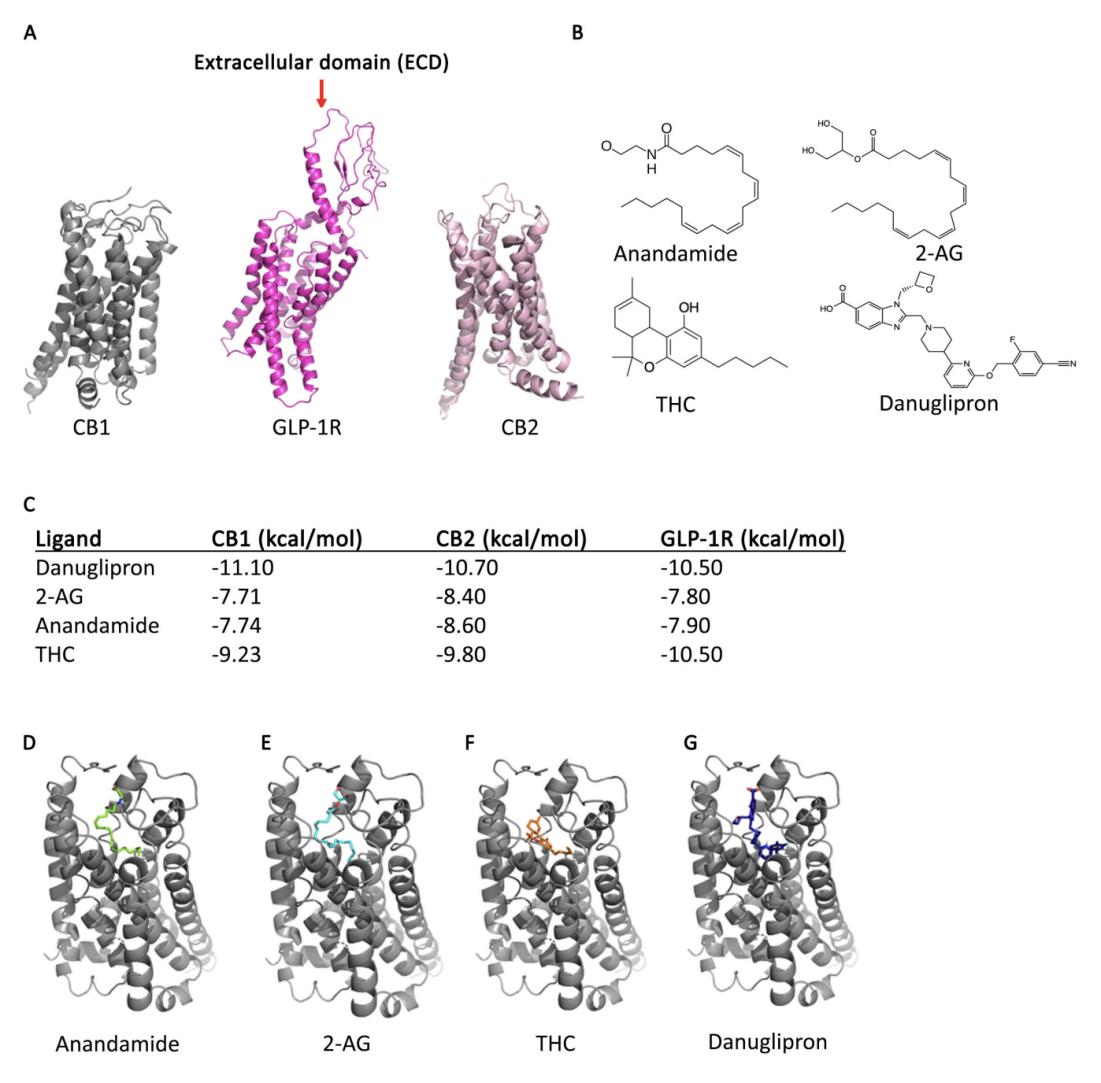
**A.** Crystal structures of CB1, GLP-1R and CB2 as reported on Protein Data Bank. **B.** Molecular structures of all ligands used in molecular docking. **C.** Binding affinities of ligand library in GLP-1R, CB1, and CB2 as calculated with SeamDock. **D.** Docking results in CB1 with anandamide (green). **E** . Docking results in CB1 with 2-AG (blue). **F** . Docking results in CB1 with THC (orange). **G** . Docking results in CB1 with danuglipron (dark blue).

## Description

The demand for so-called weight-loss drugs targeting the glucagon-like peptide-1 (GLP1) receptor has skyrocketed in recent years. According to FAIR Health, there has been well over a 300% increase in adults prescribed any type of GLP-1 drug since 2019 (FAIR Health 2025). As of May 2024, 12% of adult Americans reported currently using or having previously used a GLP-1 drug, based on data from the national polling organization KFF (Harris 2024). In parallel, but independent from the growing use of GLP-1 therapies, cannabis has also seen a rise in both prevalence and public acceptance. With a few exceptions, most US states permit some form of marijuana use (Hong, Sideris et al. 2024).

Given the accelerating, separate interest in both GLP-1-based therapies and cannabis, there is an increasing need to investigate the potential biological crossover between the two systems, specifically because both target G protein-coupled receptors (GPCRs). CB1, the primary cannabinoid receptor in the central nervous system, is a class A (rhodopsin-like) GPCR that controls the activity of neurotransmitters. CB2 is also a class A GPCR that is more prominent in immune cells but also can modulate intestinal inflammation (Lu and Mackie 2021). Class A receptors typically favor small-molecule or small-peptide ligands due to their shorter extracellular domain (ECD), and ligand binding occurs deep within the transmembrane core, directly triggering receptor activation (Vu, Bender et al. 2021).

Unlike CB1 and CB2, GLP-1R is a class B (secretin-like) GPCR characterized by a larger ECD ( **Fig.1A** ), which binds larger peptide ligands such as native GLP-1. This structural distinction contributes to a fundamentally different activation mechanism. Class B GPCRs follow a two-domain binding model: the ECD interacts with the C-terminal portion of the peptide ligand, while the transmembrane domain (TMD) binds the N-terminal region. This dual interaction promotes closure of the extracellular loops, stabilizing the receptor in its active conformation (Gonzalez-Mariscal, Krzysik-Walker et al. 2016).

GLP-1 is a hormone secreted by L-cells in the small intestine in response to nutrient intake. It stimulates insulin secretion, enhances glucose uptake via upregulation of glucose transporters, and suppresses appetite, thereby promoting blood glucose homeostasis and reducing food intake (Knudsen, Kiel et al. 2007). These effects make GLP-1 a key target in the treatment of type 2 diabetes and obesity. Despite its therapeutic potential, the clinical use of native GLP-1 is limited by its very short half-life and dose-limiting side effects, such as nausea and gastrointestinal discomfort. To address these issues, researchers have developed GLP-1 analogs with longer half-lives and more favorable pharmacokinetic properties. However, since these analogs are peptides, they must be administered via injection (Knudsen, Kiel et al. 2007), which can reduce patient compliance. Moreover, nausea and other GI-related side effects remain a challenge, even with improved peptide analogs. To overcome these limitations, alternative strategies have been explored, including targeting the endocannabinoid system. Different studies have uncovered potential ties between the endocannabinoid system and the GLP-1R pathway (Cheng, Ho et al. 2015, Gonzalez-Mariscal, Krzysik-Walker et al. 2016, Zizzari, He et al. 2021). CB1 receptor inhibition has been reported to enhance GLP-1R-mediated insulin secretion, and dual-target treatments combining CB1 inhibition with GLP-1R agonism have resulted in accelerated weight loss in mice compared to GLP-1R agonists alone (Gonzalez-Mariscal, Krzysik-Walker et al. 2016, Zizzari, He et al. 2021). Additionally, some ligands with structural similarities to endocannabinoids—referred to as endocannabinoid-like ligands—have been shown to increase the potency of some GLP-1 drugs, helping to regulate GLP-1R signaling (Cheng, Ho et al. 2015).

Pfizer has developed danuglipron (PF-06882961), an oral small-molecule GLP-1R agonist. Danuglipron has demonstrated weight loss effects comparable to injectable GLP-1 analogs while offering the advantage of oral administration and potentially reduced gastrointestinal side effects, as it is designed to be compatible with the digestive system ( **Fig. 1B** ) (Saxena, Gorman et al. 2021). The development of this small molecule introduced a possible novel allosteric activation mechanism for GLP-1R. Cryo-EM structural studies of the GLP-1 receptor (GLP-1R) bound to a danuglipron analog, PF- 06883365, revealed that the compound binds deep within a pocket formed by transmembrane helices 1, 2, 3, and 7, distinct from the traditional two-domain binding model of native GLP-1 (Griffith, Edmonds et al. 2022). Furthermore, danuglipron interacts with W33, a critical residue in transmembrane domain 1 (TMD1), as well as with extracellular loops 1 and 2 (ECL1 and ECL2), forming hydrogen bonds that help stabilize the binding pocket (Saxena, Gorman et al. 2021). PF-06883365, which has a higher binding affinity than danuglipron, enabled a more resolved cryo-EM structure, offering improved insights into danuglipron’s binding mechanism and location (Griffith, Edmonds et al. 2022). However, this finding raises the prospect of potential cross-reactivity with class A GPCRs, such as CB1 and CB2, due to their preference for binding small molecules instead of large peptides. Among roughly 500 drug candidates targeting class A GPCRs, only 134 are peptides—the majority are small molecules (Yang, Zhou et al. 2021).

We carried out molecular docking experiments to identify potential crossovers between the endocannabinoid system and GLP-1R. We chose danuglipron along with other ligands relevant to this system, including anandamide, 2-AG, and THC ( **Fig.1B** ). Anandamide and 2-AG are endogenous agonists of CB1 and CB2. THC, also an agonist of CB1 and CB2, must be ingested as it is an exogenous cannabinoid (Maccarrone and Finazzi-Agro 2003). The structurally similar cannabidiol (CBD), a related cannabinoid also found in cannabis, is known as an allosteric modulator of CB1. Thus, CBD was excluded from this study focused specifically on the orthosteric binding site where THC, anandamide, and 2-AG are known to bind.

To explore cross-reactivity between these systems, a cross-docking study was performed, assessing how ligands of one receptor might interact with another GPCR. Binding affinities from each docking study were computed ( **Fig.1C** ). To verify the reliability of the docking algorithm used in this study, a known CB1–agonist complex (AM11542 bound to CB1; PDB ID: 5XRA) was subjected to our docking protocol as a proof of concept. In PyMOL, the 5XRA structure was modified to isolate the CB1 receptor and the THC analog ligand for docking preparation. To maintain consistency, the same coordinate box used for all other CB1 dockings was applied. For this purpose, the 5XRA structure was aligned to the 6KPG structure used throughout this study. The original publication of the 5XRA crystal structure identified the primary interactions involved in AM11542 binding were hydrophobic and aromatic, predominantly involving Phe268, Phe379, Phe189, and Phe177, which interact with the tricyclic ring of THC. Two hydrogen bonds were also identified, formed with Ser383 and Ile267 (Hua, Li et al. 2020). Docking results from Seam Dock supported these key interactions, except for Ile267, which fell outside the 5 Å interaction range. Notably, AM11542 adopted an L-shaped conformation in both the crystal structure and the Seam Dock model, consistent with all other CB1 dockings performed in this study.

When observing the calculated affinity values for the ‘best’ ligand-CB1 binding complexes ( **Fig.1D-G)** , danuglipron (the GLP1R agonist) had an affinity for CB1 calculated to be -11.10 kcal/mol ( **Fig. 1G** ). This appears to indicate a potentially stronger binding affinity as compared to native CB1 ligands: anandamide (-7.74 kcal/mol) and 2-AG (-7.71 kcal/mol), and exogenous CB1 ligand THC (-9.23 kcal/mol). Although the binding affinities of the tested ligands with CB1 are different, when all the ligand-CB1 binding complexes were overlaid, we found that all ligands appear to be docking in similar bent conformations, creating an ‘L’ like shape, within the orthosteric site. In examining the values for the ‘best’ ligand-CB2 binding complexes, danuglipron had an affinity for CB2 of -10.70 kcal/mol. Like CB1, this also appears to indicate a stronger binding affinity as compared to known CB2 ligands; anandamide (-8.60 kcal/mol), 2-AG (-8.40 kcal/mol), and THC (-9.80 kcal/mol). In both cases, SeamDock results revealed more hydrophobic interactions and weak hydrogen bonding interactions between danuglipron and both CB1 and CB2 compared to the other ligands examined, thus contributing to the increased binding affinity. When analyzing the values for the ‘best’ ligand-GLP-1R binding complexes, danuglipron showed an affinity for GLP-1R of -10.50 kcal/mol. This is higher than either of the endogenous cannabinoid ligands, but interestingly, it is the same as THC, which also had a calculated binding affinity of -10.50 kcal/mol.

This analysis concludes that danuglipron, a proposed Pfizer GLP-1R agonist for weight loss, docked in a similar location and conformation as the native and non-native CB1/CB2 ligands. Danuglipron also had a higher calculated binding affinity than the cannabinoid ligands tested in CB1 and CB2. Furthermore, in GLP-1R, THC had the same binding affinity as danuglipron. These outcomes suggest that danuglipron, a small GLP-1R agonist, may possess the capacity to interact with components of the endocannabinoid system while THC may possess the capacity to interact with GLP1R in a manner similar to other small-molecule agonists. While binding affinity alone does not confirm cross-regulation, future research should experimentally evaluate the hypothesis-driven findings presented here, as they potentially implicate both systems. Further study could also explore the potential for small-molecule GLP1 agonists like danuglipron to interact with the allosteric binding site of CB1 and CB2.

## Methods

SeamDock docked the ligand selections and calculated potential binding complexes (Murail, de Vries et al. 2021). All ligands were entered into SeamDock using their SMILES code, and each receptor was entered as a PDB file, which was retrieved from the Protein Data Bank. The PDB ID’s were as follows: CB1(6KPG), CB2 (8GUQ), and GLP-1R (7LCJ) (Hua, Li et al. 2020, Zhang, Belousoff et al. 2021, Li, Chang et al. 2023). The same docking parameters were selected for the docking of each ligand, other than for the coordinate box size and where the center was focused. For CB1 and CB2, the coordinate box size was 20 Å x 20 Å x 20 Å. For GLP-1R, the box size was 20 Å x 25 Å x 25 Å. The center coordinates for CB1 were -3 Å, y: 8 Å, z: 7 Å, for CB2 x: 2 Å, y: -9 Å, z: -10 Å, and for GLP-1R x: 0 Å, y: 15 Å, z: 0 Å. The coordinates for where the center should be were based on the literature of where critical binding residues were found. The software was set to Vina (Eberhardt, Santos-Martins et al. 2021), spacing was kept at 1.0, mode number was set to 10, and the energy range and exhaustiveness were kept at 5 and 8. Once the parameters were set, the docking was able to run. The binding complex (in kcal/mol) that had the lowest affinity value (most negative) was deemed to be the ‘best’ complex and therefore was the binding complex that was used. The PyMOL Molecular Graphics System, Version 2.0 Schrödinger, LLC was used for analyses and receptor isolation from PDB files (Schrodinger 2020).

## References

[R1] FAIR Health, I., *Obesity and GLP-1 drugs.* White paper, 2025.

[R2] Harris E (2024). Poll: Roughly 12% of US Adults Have Used a GLP-1 Drug, Even If Unaffordable.. JAMA.

[R3] Hong G, Sideris A, Waldman S, Stauffer J, Wu CL (2023). Legal and Regulatory Aspects of Medical Cannabis in the United States.. Anesth Analg.

[R4] Lu HC, Mackie K (2020). Review of the Endocannabinoid System.. Biol Psychiatry Cogn Neurosci Neuroimaging.

[R5] Vu O, Bender BJ, Pankewitz L, Huster D, Beck-Sickinger AG, Meiler J (2021). The Structural Basis of Peptide Binding at Class A G Protein-Coupled Receptors.. Molecules.

[R6] González-Mariscal I, Krzysik-Walker SM, Kim W, Rouse M, Egan JM (2015). Blockade of cannabinoid 1 receptor improves GLP-1R mediated insulin secretion in mice.. Mol Cell Endocrinol.

[R7] Knudsen LB, Kiel D, Teng M, Behrens C, Bhumralkar D, Kodra JT, Holst JJ, Jeppesen CB, Johnson MD, de Jong JC, Jorgensen AS, Kercher T, Kostrowicki J, Madsen P, Olesen PH, Petersen JS, Poulsen F, Sidelmann UG, Sturis J, Truesdale L, May J, Lau J (2007). Small-molecule agonists for the glucagon-like peptide 1 receptor.. Proc Natl Acad Sci U S A.

[R8] Zizzari P, He R, Falk S, Bellocchio L, Allard C, Clark S, Lesté-Lasserre T, Marsicano G, Clemmensen C, Perez-Tilve D, Finan B, Cota D, Quarta C (2020). CB1 and GLP-1 Receptors Cross Talk Provides New Therapies for Obesity.. Diabetes.

[R9] Cheng YH, Ho MS, Huang WT, Chou YT, King K (2015). Modulation of Glucagon-like Peptide-1 (GLP-1) Potency by Endocannabinoid-like Lipids Represents a Novel Mode of Regulating GLP-1 Receptor Signaling.. J Biol Chem.

[R10] Saxena AR, Gorman DN, Esquejo RM, Bergman A, Chidsey K, Buckeridge C, Griffith DA, Kim AM (2021). Danuglipron (PF-06882961) in type 2 diabetes: a randomized, placebo-controlled, multiple ascending-dose phase 1 trial.. Nat Med.

[R11] Griffith DA, Edmonds DJ, Fortin JP, Kalgutkar AS, Kuzmiski JB, Loria PM, Saxena AR, Bagley SW, Buckeridge C, Curto JM, Derksen DR, Dias JM, Griffor MC, Han S, Jackson VM, Landis MS, Lettiere D, Limberakis C, Liu Y, Mathiowetz AM, Patel JC, Piotrowski DW, Price DA, Ruggeri RB, Tess DA (2022). A Small-Molecule Oral Agonist of the Human Glucagon-like Peptide-1 Receptor.. J Med Chem.

[R12] Yang D, Zhou Q, Labroska V, Qin S, Darbalaei S, Wu Y, Yuliantie E, Xie L, Tao H, Cheng J, Liu Q, Zhao S, Shui W, Jiang Y, Wang MW (2021). G protein-coupled receptors: structure- and function-based drug discovery.. Signal Transduct Target Ther.

[R13] Maccarrone M, Finazzi-Agró A (2003). The endocannabinoid system, anandamide and the regulation of mammalian cell apoptosis.. Cell Death Differ.

[R14] Murail S, de Vries SJ, Rey J, Moroy G, Tufféry P (2021). SeamDock: An Interactive and Collaborative Online Docking Resource to Assist Small Compound Molecular Docking.. Front Mol Biosci.

[R15] Hua T, Li X, Wu L, Iliopoulos-Tsoutsouvas C, Wang Y, Wu M, Shen L, Brust CA, Nikas SP, Song F, Song X, Yuan S, Sun Q, Wu Y, Jiang S, Grim TW, Benchama O, Stahl EL, Zvonok N, Zhao S, Bohn LM, Makriyannis A, Liu ZJ (2020). Activation and Signaling Mechanism Revealed by Cannabinoid Receptor-G(i) Complex Structures.. Cell.

[R16] Li X, Chang H, Bouma J, de Paus LV, Mukhopadhyay P, Paloczi J, Mustafa M, van der Horst C, Kumar SS, Wu L, Yu Y, van den Berg RJBHN, Janssen APA, Lichtman A, Liu ZJ, Pacher P, van der Stelt M, Heitman LH, Hua T (2023). Structural basis of selective cannabinoid CB(2) receptor activation.. Nat Commun.

[R17] Zhang X, Belousoff MJ, Liang YL, Danev R, Sexton PM, Wootten D (2021). Structure and dynamics of semaglutide- and taspoglutide-bound GLP-1R-Gs complexes.. Cell Rep.

[R18] Eberhardt J, Santos-Martins D, Tillack AF, Forli S (2021). AutoDock Vina 1.2.0: New Docking Methods, Expanded Force Field, and Python Bindings.. J Chem Inf Model.

[R19] Schrodinger, L.D., W, *PyMOL.* Retrieved from http://www.pymol.org/pymol, 2020.

